# Identification of a Novel Mutation of β-Spectrin in Hereditary Spherocytosis Using Whole Exome Sequencing

**DOI:** 10.3390/ijms222011007

**Published:** 2021-10-12

**Authors:** Dżamila M. Bogusławska, Michał Skulski, Beata Machnicka, Stanisław Potoczek, Sebastian Kraszewski, Kazimierz Kuliczkowski, Aleksander F. Sikorski

**Affiliations:** 1 Department of Biotechnology, Institute of Biological Sciences, University of Zielona Góra, Prof. Szafrana St. 1, 65-516 Zielona Góra, Poland; d.boguslawska@wnb.uz.zgora.pl (D.M.B.); b.machnicka@wnb.uz.zgora.pl (B.M.); 2 Department of Cytobiochemistry, Faculty of Biotechnology, University of Wrocław, ul. Fryderyka Joliot-Curie 14a, 50-383 Wrocław, Poland; michal.skulski@uwr.edu.pl; 3 Department and Clinic of Haematology, Blood Neoplasms, and Bone Marrow Transplantation, Wroclaw Medical University, Wybrzeże L. Pasteura 4, 50-367 Wroclaw, Poland; stanislaw.potoczek@umed.wroc.pl; 4 Department of Biomedical Engineering, Wrocław University of Science and Technology, Plac Grunwaldzki 13 (D-1), 50-377 Wrocław, Poland; sebastian.kraszewski@pwr.edu.pl; 5 Silesian Park of Medical Technology Kardio-Med Silesia, ul. M. Curie-Skłodowskiej 10c, 41-800 Zabrze, Poland; kazimierz.kuliczkowski@umed.wroc.pl; 6 Research and Development Centre, Regional Specialist Hospital, Kamieńskiego 73a, 51-154 Wroclaw, Poland

**Keywords:** hereditary spherocytosis, β-spectrin, actin binding domain, whole exome sequencing

## Abstract

Hereditary spherocytosis (HS), the most commonly inherited hemolytic anemia in northern Europeans, comprises a group of diseases whose heterogeneous genetic basis results in a variable clinical presentation. High-throughput genome sequencing methods have made a leading contribution to the recent progress in research on and diagnostics of inherited diseases and inspired us to apply whole exome sequencing (WES) to identify potential mutations in HS. The data presented here reveal a novel mutation probably responsible for HS in a single Polish family. Patients with clinical evidence of HS (clinical symptoms, hematological data, and EMA test) were enrolled in the study. The examination of the resulting WES data showed a number of polymorphisms in 71 genes associated with known erythrocyte pathologies (including membranopathies, enzymopathies, and hemoglobinopathies). Only a single *SPTB* gene variant indicated the possible molecular mechanism of the disease in the studied family. The new missense mutation p.C183Y was identified using WES in the *SPTB* gene, which is most likely the cause of clinical symptoms typical of hereditary spherocytosis (membranopathy) due to structural and functional impairments of human β-spectrin. This mutation allows for a better understanding of the molecular mechanism(s) of one of the membranopathies, hereditary spherocytosis.

## 1. Introduction

Hereditary spherocytosis (HS) is one of the most frequently inherited congenital hemolytic anemias in Caucasians with a prevalence of approximately 1 in 2000 births [1,2]. This erythrocyte membranopathy is a clinically, biochemically, and genetically heterogeneous disorder in which the presenting phenotype is anemia (which can range from compensated to severe), jaundice, reticulocytosis, splenomegaly, gallstones, several spherocytes in a peripheral blood smear, and increased erythrocyte osmotic fragility [3,4]. Previous studies have shown that five genes (*ANK1*, *SPTB*, *SPTA1*, *SLC4A1,* and *EPB42*) encoding red blood cell (RBC) membrane or skeletal proteins are mutated in hereditary spherocytosis [5,6]. A deficiency in or the dysfunction of these proteins results in a reduced surface area-to-volume ratio of spheroidal-shaped red cells due to membrane loss (mostly the intrinsic protein-containing lipid bilayer) and decreased membrane mechanical stability and deformability, which are responsible for the selective trapping of these cells in the spleen [7,8].

Known HS mutations deposited in the Human Genome Mutation Database (HGMD) are usually unique to selected families, but they allow for a better understanding of the nature of vertical interactions between the membrane skeleton and the integral protein-containing lipid bilayer. In general, spectrin deficiency is frequently identified as the causative factor in hereditary spherocytosis (OMIM, # 616649 and # 182900). Cases with autosomal recessive inheritance are caused by defects in α-spectrin, whereas dominant inheritance is associated with β-spectrin gene defects or secondary to ankyrin gene defects [9,10,11,12]. This different pattern of inheritance of hereditary spherocytosis for the *SPTA1* and *SPTB* genes is a consequence of the differences in regulation of expression of these proteins. Under physiological conditions, the expression level of the former of these genes is three to four times higher than that of the latter. Therefore, mutations of only one allele of the *SPTB* gene are sufficient for the manifestation of clinical symptoms of HS, as these mutations lead to the production of a nonfunctional spectrin or β-spectrin deficiency. It is estimated that mutations in the *SPTB* gene correlating with the HS phenotype account for 15%–30% of cases reported in the northern European population [2]. Literature data characterizing the Polish population confirm these estimates, although reports on this subject are still scarce [13,14,15,16,17,18]. Several characterized cases involved mutations in the *SPTB* gene [16,17]. In addition, in a single family, a defect in the gene encoding erythrocyte ankyrin was detected in HS patients in three generations [18]. Defects in spectrin reduce the density of the membrane skeleton, destabilizing membrane structural integrity, which is also responsible for the generation and release of microvesicles, a process that leads to the mentioned membrane loss [2,19]. In contrast to the other major group of hemolytic anemia cases with defects in the *SLC4A1* gene encoding anion exchanger 1 (band 3, AE1), these microvesicles contain AE1. In severe HS cases, splenectomy is usually recommended, but its effectiveness in relieving symptoms of anemia depends on the type of defect. Better results are achieved in patients with spectrin/ankyrin defects than in those with AE1-deficient erythrocytes, which after release of microvesicles contain a relative excess of skeletal proteins [20]. HS patients respond clinically to splenectomy, but the underlying abnormalities in erythrocytes persist, and thus the circulation lifespan, although markedly increased, is still reduced compared with normal. This is usually compensated for by increased erythropoiesis, which can be observed as an increased reticulocyte count. Splenectomy is also effective in the treatment of a group of similar membranopathies, such as hereditary elliptocytosis, while it is not recommended for two other membranopathies—dehydrated hereditary stomatocytosis and hereditary overhydrated stomatocytosis [8,21].

Over the last decade, significant advances have been made in diagnosis and clinical management of hereditary spherocytosis and other erythrocyte diseases [22,23,24,25]. Diagnostics of hereditary spherocytosis include several research methods that are still being improved [3,26,27]. The most sensitive methods used to distinguish HS from other hemolytic anemias are: the eosin-5′-maleimide (EMA) binding test, the acidified glycerol lysis test (AGLT), SDS-PAGE electrophoresis, and molecular diagnostics [4]. Among the biochemical tests, the eosin-5′-maleimide (EMA) binding test is the most frequently recommended and the most sensitive [3,4,26,28]. The proper diagnosis is difficult to verify for patients with combined occurrence of various diseases. New literature data have significantly enriched the list of known HS-related mutations, and also allowed for the verification and correction of diagnosis of previously diagnosed cases of hemolytic anemias [29,30,31,32,33]. As the research of Russo et al. shows, the initial clinical diagnosis was changed in 45.8% of patients with hemolytic anemia [29]. This mainly concerned cases pre-diagnosed as congenital dyserythropoietic anemia (CDA). At the same time, the diagnosis of HS was confirmed in all analyzed cases.

The development of high-efficiency genome sequencing methods such as whole exome sequencing (WES) gives new diagnostic opportunities and makes it possible not only to identify new mutations, but also to determine the novel causative genes [34]. Here, we report a hitherto unknown missense mutation (p.C183Y) in the β-spectrin gene, which was detected using WES and confirmed by standard Sanger sequencing. It was correlated with the HS phenotype in a two-generation Polish family with autosomal dominant HS. The newly discovered mutation in the *SPTB* gene is most likely the cause of clinical symptoms typical of one of the membranopathies, hereditary spherocytosis.

## 2. Results

Our work started with the analysis of the clinical symptoms and laboratory data, followed by the WES analysis, a detailed study of all detected defects, and further confirmation of potentially significant variants by Sanger sequencing of gDNA and cDNA. We then analyzed information about known mutations of this gene correlated with the HS phenotype as well as the structure and function of the actin binding domain of β-spectrin. Finally, we attempted to explain the functional consequences of the newly detected mutation in the *SPTB* gene.

### 2.1. Case Presentation

Patients originating from Poland with clinical evidence of hereditary spherocytosis were enrolled for WES analysis. The two family members with moderate symptoms of the disease, a mother (J42, age 54, unsplenectomized) and her daughter (J41, age 26, splenectomized and cholecystectomized in her childhood, at the age of 9), were patients of the clinic of Wroclaw Medical University, Department and Clinic of Haematology, Blood Neoplasms, and Bone Marrow Transplantation. Other members of the family, in particular the father of J41, reported as clinically normal, were unavailable. Diagnostic criteria for HS were based on typical features: spherocytes on peripheral blood smears, splenomegaly, increased bilirubin, reticulocytosis, anemia (Table 1), and increased osmotic fragility of RBCs (minimal resistance of cells—100 mmol NaCl/L for the splenectomized daughter and 87 mmol NaCl/L for the unsplenectomized mother; reference value: 78 mmol NaCl/L). A lack of a spleen results in longer erythrocyte survival, a higher number of them in the peripheral blood, and, consequently, a greater increase in the osmotic fragility of RBCs. The direct antiglobulin test was negative. Glucose-6-phosphate isomerase, phosphofructokinase, glucose-6-phosphate dehydrogenase, and pyruvate kinase activity values were normal. The clinical symptoms of the mother were much more severe, which is most likely related to the daughter’s splenectomy. The Ethics Committee of Wroclaw Medical University approved the study (protocol KB-199/2017). Informed consent was obtained from both patients before entering the protocol.

Data concerning the hematological parameters of the two related patients of the studied family are shown in Table 1. The RBC count, Hb level, reticulocytosis, and, above all, EMA test show typical HS values. These data indicate moderate HS of a heterozygous background. The parameters of the splenectomized daughter’s blood indicate near normal levels.

### 2.2. WES Results Analysis

High-quality DNAs were isolated from whole blood cells (Additional File S1, Table S1, and Section 4) and analyzed using paired-end exome sequences on the Illumina NextSeq500 platform (whole exome sequencing statistics showed that the WES had a depth of coverage of 57.18 to 57.84). More than 99% of the DNA sequences mapped to specific genomic regions. Over 99% of the reads were properly paired, indicating that both forward and reverse reads were correctly oriented. Percent duplication ranged from 18.23% to 19.07%. These parameters prove the reliability and validity of the WES data obtained (Additional File S1, Table S2, and Section 4).

Briefly, a total of 51,256 nucleotide sequence changes present in the genetic material of both patients were detected. We focused on functional variants with a potentially large effect (missense, nonsense, splicing site, and frameshift), which allowed us to select 9216 variants (Table 2). All new and low-frequency changes were thoroughly analyzed. Variants were filtered for relevance to a human disease based on population frequency (allele frequency in the 1000 Genome Project and Exome Aggregation Consortium (ExAC)) and whether they were linked to hereditary hemolytic anemia in the Online Mendelian Inheritance in Man (OMIM) database. Potentially significant genetic changes were compared with ClinVar (http://www.ncbi.nlm.nih.gov/clinvar, accessed on 27 October 2020), the Human Gene Mutation Database (http://www.hgmd.cf.ac.uk, accessed on 27 October 2020), and the Red Cell Membrane Disorder Mutations Database (http://research.nhgri.nih.gov/RBCmembrane, accessed on 27 October 2020) as well as literature data.

No known pathogenic mutations or functional variants with a potentially large effect (except one in the *SPTB* gene, p.C183Y, a variant absent in the population databases) in both patients’ genomes that may be a direct cause of the clinical HS manifestation observed in the studied family were detected. We also reached similar conclusions through analysis of twenty variants detected in genes that were described in OMIM as pathogenic, as well as variants automatically annotated by the GEMINI software program [35] as associated with anemia (for details, see Additional File S2). A missense mutation in the *SPTB* gene (p.C183Y) discovered through WES was automatically classified as a variant of unknown significance (VUS; PM2, PP3). The following functional consequences due to the p.C183Y mutation using the Ingenuity Variant Analysis plugin (IVA; QIAGEN, CA, USA) were predicted: SIFT Function Prediction—Damaging; SIFT Score—0.00; CADD Score—29.500; and Conservation phyloP *p*-value—5.284E-10 (Additional File S3).

The only new and most likely important pathogenic mutations of the *SPTB* gene (p.C183Y) were detected within the panel of 71 genes recommended for RBC pathologies by Russo et al. (Table 2; for details, see Additional File S1, Table S3, and Additional File S2) [22] and confirmed via Sanger sequencing of gDNA and cDNA (Additional File S1 and Section 4). Thirteen new variants were detected in the genes included in this panel, of which only four were present in both patients (including the above-mentioned *SPTB* gene mutation). The missense mutation of the *GATA2* gene (detected only in one patient) marked as probably damaging does not seem clinically significant. In this case, the exclusion from further analysis of this change is also due to the fact that this patient does not show the clinical symptoms described in cases of *GATA2* gene defects (myelodysplastic syndrome) [36]. The panel also contained 24 low-frequency variants (<0.03%, with the (population-independent) raw allele frequency of the variant based on ExAC exomes (AF)), but only six of them in both patients in a heterozygous state were found (Additional File S1, Table S4). However, considering the biological function of the proteins encoded by these genes, the clinical description of diseases caused by the mutation of individual genes, and the impact on severity, these variants could probably be excluded as clinically relevant for the studied case. An expanded examination of the sequence data including remaining genes related to erythrocyte membranopathies showed that the detected variants of genes associated with known RBC pathologies did not appear to cause the clinical symptoms observed in the studied patients (for details, see Additional File S2). It should be noted that, among the variants detected in the above-mentioned group of genes, the only potentially significant change was detected in the *PIEZO1* gene; namely, a single, rare polymorphism, a heterozygous splice donor variant (rs199524784, NM_001142864.3:c.1107+1G>C), was identified by the Sanger method in the genomic DNA of both patients (Additional File S1, Figure S1). However, experimentally obtained mRNA transcript sequences (Sanger method) match the human reference sequence, probably precluding a correlation of this polymorphism with the reported case.

**Figure 1:**
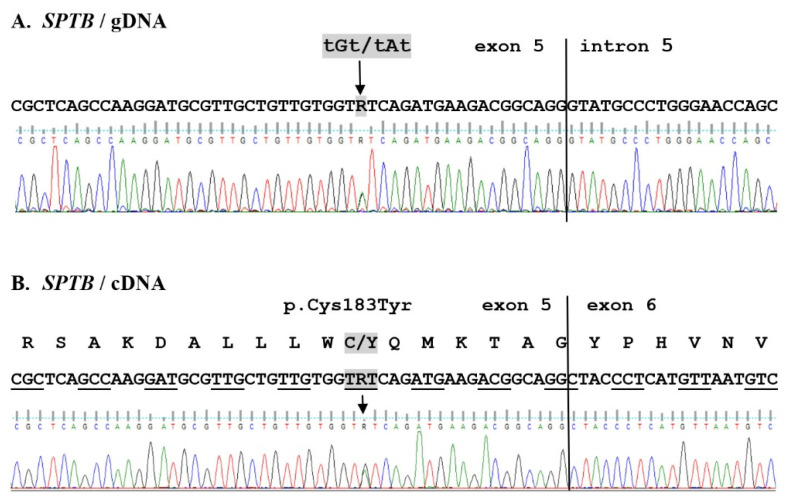
New *SPTB* mutation associated with hereditary spherocytosis. Fragment of sequencing traces of the *SPTB* gene in an affected patient (J42). Both gDNA and cDNA sequence analysis revealed a heterozygous substitution tGt/tAt in both affected patients, causing the missense mutation (p.C183Y).

Particularly interesting for us were those genes associated with the pathogenesis of HS. Analyses were focused on the five genes best known to be related to the HS phenotype (*ANK1*, *SPTB*, *SPTA1*, *SLC4A1*, and *EPB42*) (for details, see Additional File S2). Verifying the variants of the *SPTB* gene detected in the patients’ whole exome sequence led us to identify in gDNA and cDNA the novel heterozygous missense mutation p.C183Y (NM_001355436.2:c.715G>A) in exon 5 of the *SPTB* gene in both patients (Figure 1). No variants were found in the *EPB42* gene. All changes detected in the *ANK1* and *SPTA1* genes have already been published (SNP NCBI). Their frequency, inheritance pattern, and information contained in the ClinVar database allowed us to exclude their involvement in the studied HS phenotype. Eight new changes and one known change (located close to each other) were detected in the *SLC4A1* gene in patients J41 and/or J42 (Additional File S1, Figure S2A). These changes are located in intron 11 near exon 11 (NC_000017.10:g. 42331825-42331844, NC_000017.11:g.44254456-44254476). Verification of these changes in gDNA, however, did not confirm the occurrence of these changes in the studied genomes (Additional File S1, Figure S2A,B). Furthermore, experimentally validated mRNA transcript sequences match the human reference sequence (Additional File S1, Figure S2C). During DNA analysis, several common polymorphisms were validated using the Sanger method (Additional File S1, Table S5).

## 3. Discussion

In this article, we present a Polish family with a novel missense mutation in the *SPTB* gene. In the studied family members, originally assigned as patients having hereditary spherocytosis, analysis of clinical symptoms, hematological data, and EMA test results are consistent with WES data. It should also be noted that due to the severe clinical condition of the child, splenectomy and cholecystectomy were performed in the daughter (J41). As in most HS patients this procedure brought about a positive response, in contrast to her unsplenectomized mother, whose clinical condition is steadily worsening.

In search of the molecular mechanism underlying this case of HS, we decided to use the whole exome sequencing method. Our WES-based analyses did not identify variants that are present in the HDMD database and that could cause anemia in our patients. The research procedure used here was based primarily on the analysis of genes encoding erythrocyte membrane proteins, in particular a panel recommended for hemolytic anemias suggested by the literature data [4,29,34]. Among the new variants detected in this panel, only one in the *SPTB* gene (the heterozygous missense mutation p.C183Y) was marked as clinically significant (CADD Score 29.500) and was detected in both patients. The presence of this change was confirmed by the Sanger method in the gDNA and cDNA of both patients. It should be mentioned that functional SIFT analysis confirmed a high probability of linkage of this mutation to the observed case. A more detailed discussion is provided below.

Heterozygous changes found originally in WES in *SLC4A1* and *PIEZO1* genes, although confirmed via Sanger sequencing of gDNA, were not found in the transcripts of these genes, as their sequences were identical to the reference human sequence. In addition, it is known that the identified change in the *PIEZO1* gene gDNA is associated with autosomal dominant dehydrated hereditary stomatocytosis (DHSt). However, it probably does not appear to be the case here, as a positive response to splenectomy and a lack of thromboembolic complications after splenectomy in patient J41, together with the absence of stomatocytes and normal iron and ferritin levels in both patients [37,38,39,40], were observed.

In conclusion, analysis of the sequence data of HS-related genes revealed a new heterozygous missense mutation (p.C183Y) in the *SPTB* gene in a family with autosomal dominant HS. To date, five β-spectrin missense mutations that disrupt the N-terminal actin binding domain (ABD), including four that have been reported in human HS (p.M1V, p.W182G, p.W202R, and p.I220V), have been deposited in the HGMD [41,42,43]. The stability and elasticity of the membrane are strictly regulated by the complex of at least five major proteins: spectrin, actin, protein 4.1R, anion exchanger 1, and ankyrin-1 [44,45,46]. One of the above-mentioned mutations in the *SPTB* gene (p.W202R), which was previously described by Becker et al. [41], causes defective protein 4.1 binding due to spontaneous degradation of the mutated protein. According to the authors of this discovery, a mutated amino acid residue (tryptophan) is critical for protein 4.1 binding activity. On the other hand, another mutation cited above (p.W182G) [42] was located close to the p.C183Y mutation reported here. Both adjacent amino acid residues are highly conserved among the two CH domains’ actin binding proteins [44,47]. Therefore, the association of these two missense mutations with the observed HS phenotype is highly probable. The actin binding domain of spectrin contains two calponin homology (CH1 and CH2, residues 51–156 and 171–282, respectively) domains [48]. As previously demonstrated, these two subdomains separately bind protein 4.1 and f-actin. However, deletion of the region of the first 20 amino acid residues of the CH2 domain (171–190) significantly increases its interactions with both protein 4.1 and f-actin, indicating that these 20 residues prevent binding of CH2 to actin and protein 4.1 [49]. An interesting hypothesis was proposed by researchers investigating the α-actinin actin binding domain [50], whose elegant structural study led to the conclusion that tandem CH domains bind f-actin in an open conformation, so they have to spatially separate or to structurally rearrange from the initial closed conformation to bind actin. When both domains are active and close to each other, the steric clash prevents binding. We could speculate that also in the case of β-spectrin’s actin binding domain, existence of both active CH1 and CH2 would result in a steric clash preventing binding of f-actin and also protein 4.1, thus explaining the role of the above-mentioned “inhibitory” helix. In our opinion, this could explain the pathogenicity of the reported new missense mutation (p.C183Y) and also previously reported mutations.

## 4. Materials and Methods

### 4.1. Hematological Parameters

All hematological laboratory tests for the evaluation of anemia and RBC abnormalities were performed by professional medical laboratories (mainly during hospitalization of patients). The osmotic fragility test of RBCs was determined to measure erythrocyte resistance to hemolysis in the standard unincubated test by measuring the degree of hemolysis in hypotonic saline solution. The EMA test was performed by the Institute of Hematology and Transfusion Medicine (Warsaw, Poland).

### 4.2. DNA Isolation

EDTA preserved blood was collected from both patients (J41 and J42) by venipuncture. Genomic DNA was isolated from whole blood from both J family members using the standard method (QIAamp DNA Blood Mini Kit, Qiagen, Hilden, Germany) and stored at −20 °C until analysis. Purified DNA concentration and quality were determined by absorbancy in a UV spectrophotometer (Table S1).

### 4.3. Whole Exome Sequencing 

DNA samples were whole exome sequenced at the Heflin Center for Genomic Science Core Laboratories, University of Alabama at Birmingham (UAB), AL, USA. Whole blood DNA was subject to exome capture, performed using the Agilent SureSelect Human Clinical Research Exome (CRE) capture kit (Agilent Technologies, Inc., Santa Clara, CA, USA). The bioinformatic analysis for detecting single nucleotide variants (SNVs) and inserts/deletions was performed at the Heflin Center at UAB using the GEMINI (v0.19.1) software program [35] and annotated according to dbSNP. The Ingenuity Variant Analysis plugin (IVA; QIAGEN, Redwood City, CA, USA) was used for further filtering, annotation, and interpretation of detected variants.

Exome sequencing was performed for both affected patients. The SureSelect Target Enrichment System (Agilent Technologies, Inc., Santa Clara, CA, USA) was used, followed by 75 bp paired-end sequencing on an Illumina NextSeq500 (Illumina, Inc., San Diego, CA, USA). Raw sequence reads were aligned to the reference human genome (human genome 19/GRCh37.13).

### 4.4. Mutation Validation 

Potentially pathogenically significant variants selected by whole exome sequencing were confirmed by targeted genomic DNA and cDNA Sanger sequencing. Total RNA was extracted from whole blood and collected using the miRNeasy Mini Kit (Qiagen, Hilden, Germany) and reversely transcribed into cDNA for sequencing using the RevertAid First Strand cDNA Synthesis Kit (Thermo Fisher Scientific, Waltham, MA, USA). Sanger sequencing was performed and all primers were synthesized by Genomed S.A. (Warsaw, Poland). The sequences of the primers used to verify the key project results are shown in Table S3. The sequences of all primers and the PCR amplification parameters are available upon request.

## 5. Conclusions

The WES-based study allowed us to report and describe the molecular basis of HS in a two-generation Polish family with autosomal dominant HS. A new missense mutation, p.C183Y, was identified in the *SPTB* gene, which is most likely the cause of clinical symptoms typical of hereditary spherocytosis. Probably, similarly to another HS mutation previously described in the literature (p.W182G), it led to the structural and functional impairments of human β-spectrin. This mutation allows for a better understanding of the nature of interactions between the membrane skeleton and the lipid bilayer as well as the genotype–phenotype correlation in HS.

## Figures and Tables

**Table 1:** Hematological characteristics of studied family members. * IF—mean fluorescence intensity; HCT—hematocrit; Hb—hemoglobin; Ret—reticulocytosis.

Study J Family Members	RBC (T/L)	HCT (L/L)	Hb (mmol/L)	Total Bilirubin (µmol/L)	Direct Bilirubin (µmol/L)	Indirect Bilirubin (µmol/L)	Ret (Fraction)	EMA Test (IF) *
References for female	3.7–5.1	0.37–0.47	7.45–9.93	5.13–20.53	<5.13	1.71–17.10	0.005–0.015	98.86–117.20
J41	3.70 ± 0.10	0.35 ± 0.10	7.63 ± 0.19	11.55 ± 1.28	4.70 ± 0.43	6.84 ± 0.86	0.010 ± 0.001	90.68 ± 0.49
J42	2.95 ± 0.15	0.27 ± 0.01	5.93 ± 0.16	45.50 ± 2.22	16.93 ± 0.68	28.56 ± 1.54	0.075 ± 0.03	89.51 ± 1.29

**Table 2:** Summary of the translation impact of variant (mutations/polymorphisms) types for all variants identified in both patients with HS.

Translation Impact of Variants	Total Genes		71 Genes Involved in Known RBC Pathologies	
	Total	Not Reported	Total	Not Reported
Missense mutation	8657	45	58	2
Frameshift	248	7	2	0
In-frame	177	0	2	0
Start lost	14	0	0	0
Stop lost	29	0	0	0
Stop gained	91	2	0	0
Total variants	9216	284	398	13

## Data Availability

All data generated or analyzed during this study are included in this article and its additional files. WES datasets used and/or analyzed during this study are available from the corresponding author on reasonable request.

## Supplementary Materials

The following are available online at https://www.mdpi.com/article/10.3390/ijms222011007/s1. Additional File S1: Supplementary figures and tables. This file contains supplementary material: Figure S1: Sequencing chromatogram and expression analysis of piezo type mechanosensitive ion channel component 1 excluded the possibility of tissue-specific effects of the rare polymorphism of the *PIEZO1* gene. Figure S2: Verification of *SLC4A1* gene variants detected by the WES method in patients J41 and J42. Table S1: Quantitation of DNA isolated from whole blood cells (patients J41 and J42) indicates that DNA preparations were suitable for sequencing. Table S2: Quality control of WES raw reads. Table S3: PCR primer sequences. Table S4: Low-frequency variants (<0.03%, with the (population-independent) raw allele frequency of the variant based on ExAC exomes (AF)) occurring in a heterozygous state in both patients were detected using WES in a panel of genes having 71 genes according to the analysis conducted by Russo et al. [29]. Table S5: Polymorphisms identified during the Sanger sequencing analysis of all of the tested genes in the subjects from the studied J family. Additional File S2: Supplementary tables. Detailed data on the analyzed variants detected using WES and analyzed using the GEMINI (v0.19.1) software program. Additional File S3: Detailed data on the analyzed variants detected by WES and analyzed using the Ingenuity Variant Analysis plugin (IVA; QIAGEN, CA, USA).
