# Supplementary material for: Identification of a Novel Mutation of β-Spectrin in Hereditary Spherocytosis Using Whole Exome Sequencing
Source: Int J Mol Sci. 2021 Oct 12;22(20):11007. doi: 10.3390/ijms222011007 (PMC8540824; doi:10.3390/ijms222011007)
Supplement: Supplementary file 1 [file ijms-22-11007-s001.zip › ijms-1388284_BogusławskaetalAdditfile1.pdf]

Supplementary Materials for

Identification of novel mutation of β-spectrin in hereditary spherocytosis with help of whole exome sequencing

<u>Additional file S1</u>

Supplementary Figure legends

Figure S1.

Sequencing chromatogram and expression analysis of piezo type mechanosensitive ion channel component 1 excluded the possibility of tissue-specific effects of the rare polymorphism of the *PIEZO1* gene. **A.** Sequencing of the J42 patient gDNA showing the heterozygous substitution C→G (rs199524784) in the first nucleotide in the intron 9 of the *PIEZO1* gene. **B.** cDNA sequence of the J42 patient showing the wild type base at the junction of exons 9 and 10 of the *PIEZO1* gene.

Figure S2. Verification of *SLC4A1* gene variants detected by WES method in patients J41 and J42. Sequence analysis showed that the sequence of the analyzed fragment of gDNA and cDNA are fully consistent with the human reference sequence. **A.** Fragment of sequencing traces showing localization of nine variants (in frame) detected by WES in the intron 11 of the *SLC4A1* gene in an affected patient (J41) (NC_000017.10:g.42331825-42331844, NC_000017.11:g.44254456-44254476). Verified variants within this region are marked red. **B.** Alignment of gDNA sequencing reads to the human reference sequences. **C.** cDNA sequence of the J41 patient showing the wild type base at the junction of exons 11 and 12 of the *SLC4A1* gene.

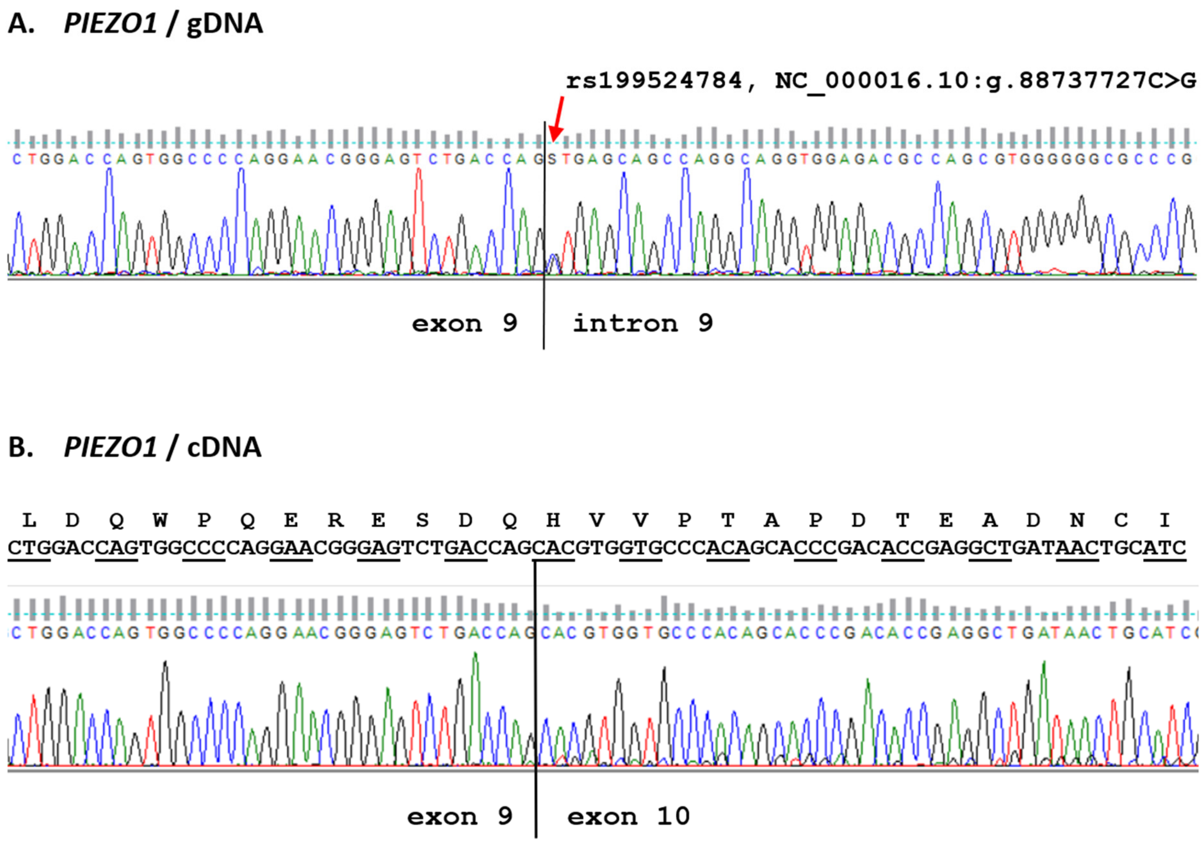
A. PIEZO1 / gDNA
rs199524784, NC_000016.10:g.88737727C>G
CTGGACCAGTGGCCCCAGGAACGGGAGTCTGACCAGSTGAGCAGCCAGGCAGGTGGAGACGCCAGCGTGGGGGGCGCCCG
exon 9
intron 9
B. PIEZO1 / cDNA
L D Q W P Q E R E S D Q H V V P T A P D T E A D N C I
CTGGACCAGTGGCCCCAGGAACGGGAGTCTGACCAGCACGTGGTGCCCACAGCACCCGACACCGAGGCTGATAACTGCATC
exon 9
exon 10

Figure S1.

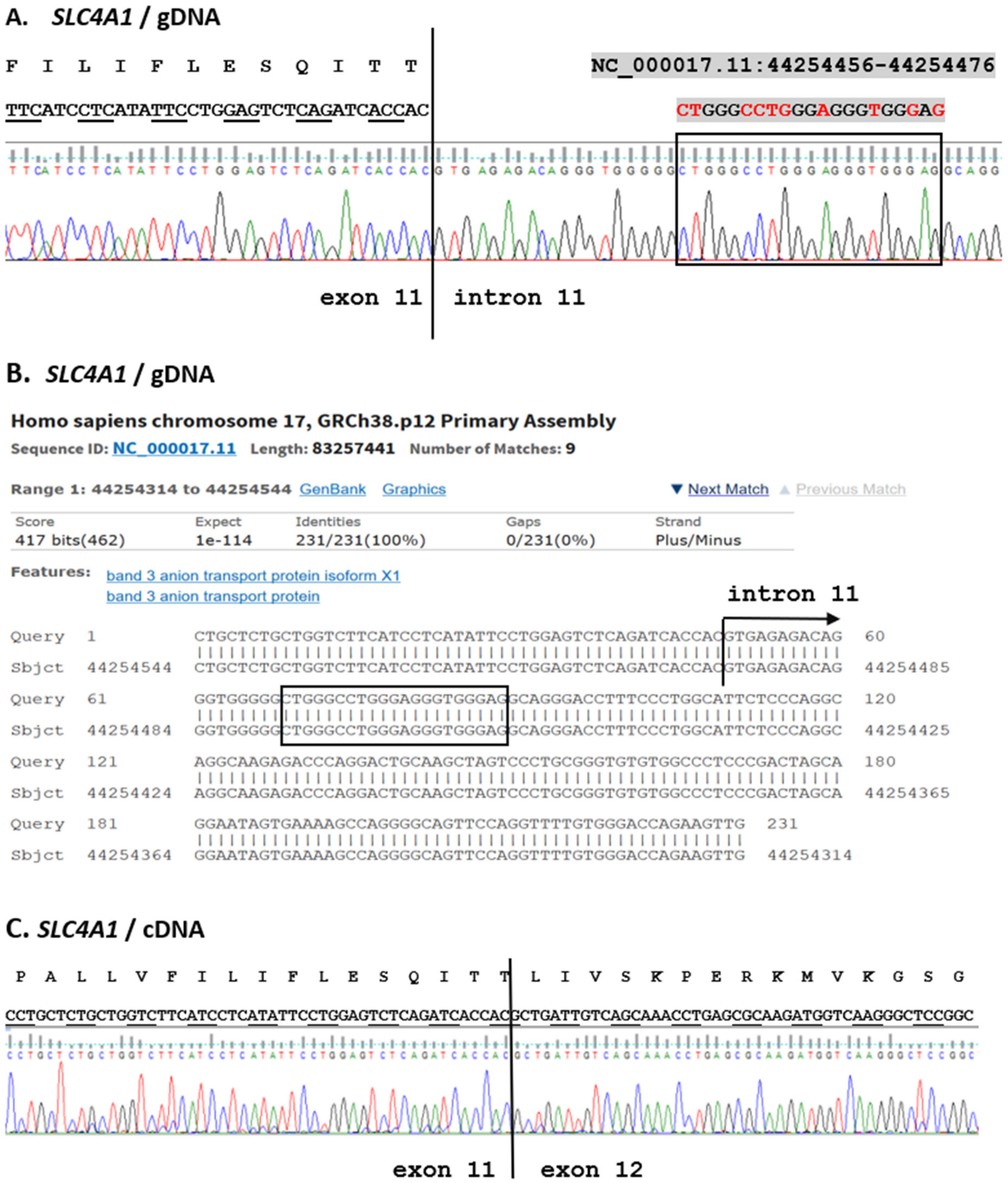
A. SLC4A1 / gDNA
F I L I F L E S Q I T T
NC_000017.11:44254456-44254476
TTCATCCTCATATTCCTGGAGTCTCAGATCACCAC
CTGGGCCTGGGAGGGTGGGAG
exon 11
intron 11
B. SLC4A1 / gDNA
Homo sapiens chromosome 17, GRCh38.p12 Primary Assembly
Sequence ID: NC_000017.11 Length: 83257441 Number of Matches: 9
Range 1: 44254314 to 44254544 GenBank Graphics
Next Match Previous Match
Score 417 bits(462)
Expect 1e-114
Identities 231/231(100%)
Gaps 0/231(0%)
Strand Plus/Minus
Features: band 3 anion transport protein isoform X1
band 3 anion transport protein
intron 11
Query 1 CTGCTCTGCTGGTCTTCATCCTCATATTCCTGGAGTCTCAGATCACCACGTGAGAGACAG 60
Sbjct 44254544 CTGCTCTGCTGGTCTTCATCCTCATATTCCTGGAGTCTCAGATCACCACGTGAGAGACAG 44254485
Query 61 GGTGGGGGCTGGGCCTGGGAGGGTGGGAGGCAGGGACCTTTCCCTGGCATTCTCCCAGGC 120
Sbjct 44254484 GGTGGGGGCTGGGCCTGGGAGGGTGGGAGGCAGGGACCTTTCCCTGGCATTCTCCCAGGC 44254425
Query 121 AGGCAAGAGACCCAGGACTGCAAGCTAGTCCCTGCGGGTGTGTGGCCCTCCCGACTAGCA 180
Sbjct 44254424 AGGCAAGAGACCCAGGACTGCAAGCTAGTCCCTGCGGGTGTGTGGCCCTCCCGACTAGCA 44254365
Query 181 GGAATAGTGAAAAGCCAGGGGCAGTTCCAGGTTTTGTGGGACCAGAAGTTG 231
Sbjct 44254364 GGAATAGTGAAAAGCCAGGGGCAGTTCCAGGTTTTGTGGGACCAGAAGTTG 44254314
C. SLC4A1 / cDNA
P A L L V F I L I F L E S Q I T T L I V S K P E R K M V K G S G
CCTGCTCTGCTGGTCTTCATCCTCATATTCCTGGAGTCTCAGATCACCACGCTGATTGTCAGCAAACCTGAGCGCAAGATGGTCAAGGGCTCCGGC
exon 11
exon 12

Figure S2.

Supplementary Tables

Table S1. Quantitation of DNA isolated from whole blood cells (patients J41 and J42) indicates that DNA preparations were suitable for sequencing.

Sample	DNA Concentration (ng/µl)	OD 260/280
J41	30.74	1.97
J42	53.78	1.90

Table S2. Quality control of WES raw reads. Burrows-Wheeler Aligner (BWA) statistics summarize the percentage of uniquely mapped reads, the percentage of properly paired reads, the percentage of duplicated reads and the average depth for each individual sample (J41 and J42) and for combined lanes.

Sample	Total # raw reads	% mapped to genome	% properly paired	% duplication	Avg depth	LANE
J41	22 352 873	22,283,821 (99.69%)	22,142,595 (99.06%)	7,13%	16,31	1
J41	22 027 182	21,953,965 (99.67%)	21,810,120 (99.01%)	7,06%	16,08	2
J41	22 566 710	22,499,506 (99.70%)	22,359,672 (99.08%)	7,23%	16,45	3
J41	22 214 589	22,146,819 (99.69%)	22,008,781 (99.07%)	7,16%	16,2	4
**J41**	**72 485 743**	**72,365,002 (99.83%)**	**72,049,052 (99.40%)**	**19,07%**	**57,18**	**1-4**
J42	22 539 525	22,470,359 (99.69%)	22,330,759 (99.07%)	6,81%	16,37	1
J42	22 163 724	22,090,542 (99.67%)	21,949,825 (99.03%)	6,72%	16,11	2
J42	22 760 134	22,693,448 (99.71%)	22,556,517 (99.11%)	6,87%	16,53	3
J42	22 371 276	22,303,660 (99.70%)	22,168,556 (99.09%)	6,80%	16,25	4
**J42**	**72 450 835**	**72,330,645 (99.83%)**	**72,018,345 (99.40%)**	**18,23%**	**57,74**	**1-4**

Table S3. PCR primer sequences.

Gene	Forward primer	Reverse primer
SPTBgDNA	5'-GGGAGCATCTAGGAGAGAGAAGG-3'	5'-CAGCAGCTAACCACACACAGAGG-3'
SPTBcDNA	5'-CAAGGCTCTCCAGTTCCTCAAGG-3'	5'-GAGTCCTTCAGCTTATCAAAGTCG-3'
PIEZO1gDNA	5'-TGTGACGGGTCTTCTCTGGACAGG-3'	5'-GCAGTTATCAGCCTCGGTGTCGG-3'
PIEZO1cDNA	5'-GTGCTGGGTCTCAAGGACTTCG-3'	5'-TGTGGTAGGTGATGCTCCATACC-3'
SLC4A1gDNA	5'-TGCGTTCCGAGTTTCCCATCTGG-3'	5'-CTCCAAATTATACAACTTCTGGTCC-3'
SLC4A1cDNA	5'-GATGGCTTCAAGGTGTCCAACTCC -3'	5’-CACTGATCCGCTGCTCTTTGACC -3'

Table S4. Low frequency variants (<0.03%, raw allele frequency (population independent) of the variant based on ExAC exomes (AF) occurring in a heterozygous state in both patients were detected using WES in panel of genes having 71 genes according to the analysis conducted by Russo et al. [29].

Chr	Type	rs ids	Clinvar sig	Gene	Codon change	Aa change	Impact	Frequency/ Aaf exac all
1	snp	rs138726443	pathogenic	***FLG***	Cga/Tga	R/*	stop_gained	0.003047
3	snp	rs200519575	None	***COL6A6***			splice_donor_variant	0.0004056
16	indel	rs768823392, rs1060503428	conflicting_interpretations_ of_pathogenicity	***SPG7***			splice_acceptor_variant	0.0003047
8	snp	rs145739822	None	***OXR1***			splice_donor_variant	0.002294
12	snp	rs141509089	None	***GSG1***	Caa/Taa	Q/*	stop_gained	0.003888
14	snp	rs199610585	None	***IGHV4-28***	tGg/tAg	W/*	stop_gained	0.002216
16	snp	rs199524784	None	***PIEZO1***			splice_donor_variant	0.0002351
2	snp	rs549794342	pathogenic/likely_pathogenic	***NEB***	Cga/Tga	R/*	stop_gained	0.0002177
19	snp	rs148074899	None	***EMR2***	Gga/Tga	G/*	stop_gained	0.000453
20	indel	rs543974571	None	***ZNF334***	acAGgg/acgg	TG/TX	frameshift_variant	0.0002553

Table S5. Polymorphisms identified during the Sanger sequencing analysis of all of the tested genes in the subjects from the studied J family.

Gene	SNP reference number	Genomic Placements	Consequence	Patient / Inheritance
ANK1	rs1872877	NC_000008.11:g.41728053C>T	Intron Variant	J41 - heterozygotic
	rs2304870	NC_000008.11:g.41728059G>A	Intron Variant	J41 - heterozygotic
	rs1137177	NC_000008.11:g.41706167G>A	Synonymous Variant	J41 - heterozygotic
	rs2304880	NC_000008.11:g.41702091G>A	Synonymous Variant	J41 - heterozygotic
	rs2304873	NC_000008.11:g.41725776C>T	Synonymous Variant	J41 - heterozygotic
	rs504574	NC_000008.11:g.41696410C>G	Synonymous Variant	J41 - heterozygotic
	rs2304883	NC_000008.11:g.41696317G>A	Intron Variant	J42 - heterozygotic
	rs515071	NC_000008.11:g.41661944A>G	Intron Variant	J41 - heterozygotic
	rs516946	NC_000008.11:g.41661730T>C	Intron Variant	J41 - heterozygotic
	rs6150565	NC_000008.11:g.41655068_41655081delACAGCAGAGTCTAT	3 Prime UTR Variant	J41 – absent<br>J42 - heterozygotic
	rs3063734	**J41** - NC_000008.11:g.41655130_41655131GT[10/11]<br>**J41** - NC_000008.11:g.41655130_41655131GT[11/14]	3 Prime UTR Variant	J41 – heterozygotic<br>J42 - heterozygotic
SPTB	rs61392811	NC_000014.9:g.64775449G>A	Intron Variant	J42 - heterozygotic
	rs8023246	NC_000014.9:g.64774263G>A	Intron Variant	J42 - heterozygotic
	rs92070	NC_000014.9:g.64773159C>T	Intron Variant	J42 - heterozygotic
PIEZO1	rs199524784	NC_000016.10:g.88737727C>G	Intron Variant	J41,J42 - heterozygotic
